# Novel Gene Variants in a Nationwide Cohort of Patients with Pheochromocytoma and Paraganglioma

**DOI:** 10.3390/ijms252212056

**Published:** 2024-11-09

**Authors:** Idoia Martínez de Lapiscina, Estrella Diego, Candela Baquero, Elsa Fernández, Edelmiro Menendez, Maria Dolores Moure, Teresa Ruiz de Azua, Luis Castaño, Nuria Valdés, on behalf of the Collaborative Working Group

**Affiliations:** 1 Biobizkaia Health Research Institute, Centro de Investigación Biomédica en Red de Enfermedades Raras (CIBERER), Centro de Investigación Biomédica en Red de Diabetes y Enfermedades Metabólicas Asociadas (CIBERDEM), European Reference Network on Rare Endocrine Conditions (Endo-ERN), Plaza de Cruces s/n, 48903 Barakaldo, Spain; 2 Biobizkaia Health Research Institute, Endocrinology and Nutrition Department, Cruces University Hospital, Plaza de Cruces s/n, 48903 Barakaldo, Spain; estrella.diegoperojo@osakidetza.eus (E.D.); elsa.fernandezrubio@osakidetza.eus (E.F.); 3 Biobizkaia Health Research Institute, University of the Basque Country (UPV-EHU), Plaza de Cruces s/n, 48903 Barakaldo, Spain; candela.baqueromartinez@bio-bizkaia.eus; 4 Principality of Asturias Health Research Institute, Department of Endocrinology and Nutrition, Asturias Central University Hospital, Oviedo University, CIBERER. Av. Roma s/n, 33011 Oviedo, Spain; edelangot@gmail.com; 5 Biobizkaia Health Research Institute, Endocrinology and Nutrition Department, Cruces University Hospital, UPV-EHU, Plaza de Cruces s/n, 48903 Barakaldo, Spain; mariadolores.mourerodriguez@osakidetza.eus; 6 Endocrinology Department, Urduliz Hospital, Goieta 32, 48610 Urduliz, Spain; teresa.ruizdeazuaarteche@osakidetza.eus; 7 Biobizkaia Health Research Institute, Pediatric Endocrinology Department, Cruces University Hospital, UPV-EHU, Centro de Investigación Biomédica en Red de Enfermedades Raras (CIBERER), Centro de Investigación Biomédica en Red de Diabetes y Enfermedades Metabólicas Asociadas (CIBERDEM), Endo-ERN, Plaza de Cruces s/n, 48903 Barakaldo, Spain; luisantonio.castanogonzalez@osakidetza.eus; 8 Biobizkaia Health Research Institute, Endocrinology and Nutrition Department, Cruces University Hospital, UPV-EHU, Centro de Investigación Biomédica en Red de Enfermedades Raras (CIBERER), Centro de Investigación Biomédica en Red de Diabetes y Enfermedades Metabólicas Asociadas (CI-BERDEM), Endo-ERN, Plaza de Cruces s/n, 48903 Barakaldo, Spain

**Keywords:** pheochromocytoma, paraganglioma, genetics, germline

## Abstract

Pheochromocytomas (PCCs) and paragangliomas (PGLs), denoted PPGLs, are rare neuroendocrine tumours and are highly heterogeneous. The phenotype–genotype correlation is poor; therefore, additional studies are needed to understand their pathogenesis. We describe the clinical characteristics of 63 patients with PPGLs and perform a genetic study. Genetic screening was performed via a targeted gene panel, and clinical variables were compared among patients with a positive molecular diagnosis and negative ones in both PCC and PGL cohorts. The mean age of patients with PCC was 50.0, and the mean age of those with PGL was 54.0. Disease-causing germline variants were identified in 16 individuals (25.4%), twelve and five patients with PCC and PGL, respectively. Genetically positive patients were younger at diagnosis in both cohorts. Variants in genes associated with either isolated PPGLs or syndromic forms of the disease were detected in a cohort of PPGLs. We have identified novel variants in known genes and set the importance of genetic screening to every patient with PPGLs, with a special focus on the young. A longer follow up of patients with variants in genes associated with syndromic forms is of clinical value.

## 1. Introduction

Pheochromocytomas (PCCs) and paragangliomas (PGLs), collectively referred to as PPGLs, are rare neuroendocrine tumours that arise from the chromaffin cells of the adrenal medulla and the extra-adrenal paraganglia, respectively. These tumours are mostly sporadic, and while the majority are benign [1,2], a considerable proportion can represent a malignant risk (10–40%), depending on the tumour size, location [3,4], and genomic characteristics [5,6,7]

Approximately 40% of PPGL cases are caused by germline pathogenic variants, making them the most heritable among endocrine tumours [8,9,10]. In recent years, the understanding of the molecular mechanisms behind these tumours has expanded due to the identification of various susceptibility genes [10,11,12]. Variants in these genes can result in the overexpression of the hypoxia signalling pathways (cluster 1) [5,9,10,13,14], including alterations in the gene encoding the Von Hippel–Lindau (VHL) tumour suppressor protein and in genes encoding the distinct subunits of the succinate dehydrogenase complex (SDHx), or the activation of kinase receptor signalling pathways, protein synthesis, and involvement in the maintenance of neuroendocrine identity (cluster 2) [15], comprising alterations in *RET* (Rearranged During Transfection) proto-oncogene, *NF1* (Neurofibromin 1), and *TMEM127* (Transmembrane Protein 127) [16,17]. A third group of genes (cluster 3) driven by *MAML3* (Mastermind-Like Transcriptional Coactivator 3) and *CSDE1* (Cold Shock Domain Containing E1) is related to the activation of the Wnt signalling pathway and with an increased risk of metastatic PPGLs [18].

PPGLs are highly heterogeneous in their clinical presentation and, more strikingly, the phenotype does not always predict the genotype [19,20,21]. Thus, disease management is challenging due to the absence of predictive markers, which hinders an early diagnosis and treatment, and there is a better prognosis for the patient and relatives. For several genes, germline alterations cause autosomal dominant tumour syndromes in which PCCs and/or PGLs are included in the manifestations, like *NF1* variants in neurofibromatosis type 1 (NF1) [12] and variants in SDHx that cause the hereditary PPGL syndrome [22]. In contrast, the low prevalence of newly described genes in 1–3% of PCCs, for example, germline variants in *TMEM127* [23,24] and *MAX* (MYC Associated Factor X) [25,26], do not clarify the associated phenotypic characteristics.

Next-generation sequencing (NGS) technology has emerged as a valuable tool. Targeted gene panels have a greater success rate and provide increased speed and data capacity at a significantly reduced cost compared to traditional sequencing [27,28,29,30,31]. Despite this recent progress, most of the PCCs and PGLs remain genetically unexplained [12].

Molecular characterization of all patients is essential due to the high heritability of PPGLs, the metastatic risk associated with some genes, the large number of susceptible genes involved, and the type of variant (somatic, germline, or mosaicism) [32]. An early and accurate genetic diagnosis helps with appropriate clinical follow up of the patient, but also makes better familiar genetic counselling. Thus, the aim of this work was to perform a genetic study in patients with PPGLs using a customised panel, including 16 known genes.

## 2. Results

### 2.1. Clinical Characteristics of the Patients

A total of 63 patients with a diagnosis of pheochromocytoma (49/63, 77.8%) or paraganglioma (14/63, 22.2%) were studied. The mean age of patients with PCC was 54.0 (39.0–63.0) years old and 55.1% (27/49) were female, while individuals with PGL had a mean age of 55.0 (47.2–65.7) years at diagnosis. Twenty-eight percent (4/14) of the last patients were female. The clinical features and genetic findings of the patients with PCC (patients P1 to P49) and PGL (P50 to P63) are shown in Table 1 and Table 2.

At diagnosis, 42.8% of the patients with PCC presented with high blood pressure (HBP) (>140/90 mmHg) (21/49) and were considered either as persistent HBP (30.6%, 15/49) or as paroxysmal HBP (12.2%, 6/49). A total of 6.1% of patients had palpitations (3/49), 4.1% (2/49) had headaches, 2.0% had mass effects (1/49), 2.0% had sweating (1/49), and 2.0% had haematuria (1/49). Thirty-two percent (16/49) of patients were asymptomatic. We do not have data for four of the patients with PCC. Among the PGL cohort, 64.3% (9/14) had persistent high blood pressure, and tumour mass effect was the main reason for medical referral in 50.0% (7/14) of them.

Biochemical testing in the PCC cohort revealed that 70.0% (26/37) had elevated 24 h urine metanephrine levels (mean level 1795.5 μg/day (892.2–6876.5)) and 89.0% (33/37) had elevated 24 h urine normetanephrine levels (mean level 1670.0 μg/day (1102.0–3150.0)). High urine metanephrine levels were observed in 56.0% of the patients with PGL (6/11) (mean value 1092.0 μg/day (819.0–2145.0)), while only one of the patients presented elevated normetanephrine levels (patient P55, 668 μg/day).

All the PCCs were unilateral, and 55.0% (27/49) were found in the left adrenal. All the patients underwent total adrenalectomy. The mean tumour size of PCCs was 4.6 (3.4–5.8) cm. Most of the PGLs were located in the abdominal region (50.0%, 7/14), followed by the cervical location (28.6%, 4/14), lumbar region (14.3%, 2/14), and mediastinum (7.1%, 1/14). Surgery was performed in all patients with PGLs, except in patient P63 due to the associated surgical risks. The mean tumour size of PGLs was 4.5 (2.3–5.7) cm.

At 12-month follow up, 97.0% (36/37) and 84.0% (31/37) of the patients had normalised metanephrine and normetanephrine levels, respectively. At this time, the recurrence or persistence of disease was not shown in 100.0% of the patients. Three patients (3/49, 6.1%) presented with metastatic disease later. Patient P1, harbouring a pathogenic variant in SDHB (c.286+1G>A), had liver and lymph node metastases at 4-year follow up and died 12 years after initial diagnosis. In P21, metastases were detected in the liver, lung, and bones four months after diagnosis. This patient died four months later. Patient P28 presented with liver metastases at 10-year follow up, and she is still alive. We did not identify pathogenic genetic variants in P21 and P28. At 12-month follow up, biochemical evaluation of the patients with PGL showed normal range metanephrine and normetanephrine levels. None of the patients who underwent surgery presented a recurrence or persistence of disease in the imaging tests. At the last follow up, no metastatic disease was observed.

Overall, patients with pathogenic, likely pathogenic, or VUS (variant of unknown significance) variants were younger at diagnosis in both PCC (39.0 years (30.0–50.5) vs. 56.0 years (48.2–64.5), *p* = 0.01) and PGL (26.5 years (24.0–33.2) vs. 64.0 years (60.5–69.0), *p* < 0.01) compared to genetically negative patients (Table 1). We found no significant differences between the two subgroups in PCCs and PGLs for tumour size and gender.

### 2.2. Genetic Findings in the Cohort

The genetic aetiology was identified in 25.4% (16/63) of the patients with PPGL (Table 3). Overall, we detected sixteen different germline variants in sixteen patients, eight of them described for the first time in individuals with PPGL. Among all the gene changes, those in SDHx were the most common, followed by *NF1*. We detected all the variants in heterozygosis, except one in the *CYP17A1* gene in two siblings with PCC and gonadal dysgenesis. The guidelines of the American College of Medical Genetics and Genomics (ACMG) classified thirteen variants as pathogenic or likely pathogenic and the remaining three as VUS.

#### 2.2.1. Variants Identified in SDHx

SDHx alterations were found in five patients (7.9%), one patient with PCC and four with PGLs. All the variants in *SDHB* and *SDHD* genes were classified as pathogenic. Two intronic *SDHB* variants (c.286+1G>A and c.72+1G>A) that prevent the correct splicing of the gene were identified in patients P1 and P59 (29). The first (c.286+1G>A) was detected in a young female with a PCC (P1) and has been previously reported in either PCC or PGL cases [33,43]. Similarly, the variant in P59 (c.72+1G>A) has been listed in PGLs with the same phenotype [42]. Targeted panel sequencing revealed another variant in this patient in exon 38 of the *NF1* gene (c.5423C>T; p.Thr1808Met). DNA samples were not available from parents because of death related to oropharyngeal and stomach cancer, respectively. The missense c.725G>A; p.(Arg242His) variant was identified in heterozygosis in patient P51, presenting with an abdominal PGL. This variant, previously described in nonsyndromic PCCs and PGLs [37,44], is known to reduce the enzymatic activity of the SDH complex [45]. One novel variant was noted in the coding sequence of the *SDHB* gene (c.595_604delinsGG; p.Tyr199GlyfsTer20) in patient P58 presenting first with a PGL of the organ of Zuckerkandl and later in the carotid body. Remarkably, the patient’s aunt who had an extra-adrenal tumour at the organ of Zuckerkandl was a carrier, as well as a healthy mother.

A single intronic variant was found in the *SDHD* (succinate dehydrogenase complex subunit D) gene. This novel variant (c.52+1G>A) is predicted to alter the consensus splice site (see Table S1) and was found in a 46-year-old female (P50) with a bilateral carotid body PGL. The analysed relatives of this patient were all healthy and wild type for the *SDHD* variant.

#### 2.2.2. NF1 Gene Variants

Among the sixty-three PPGLs analysed, three PCCs and one PGL harboured *NF1* genetic variants (6.3%). The previously described c.586+1G>A variant was identified in patient P7. This variant decreased the splicing efficiency, and it altered the exonic insertion, leading to a frameshift effect, and it has been described in a patient with NF type 1 [46] and more recently in a PCC [35]. Our female patient was diagnosed with PCC at age 36 and currently presents a Graves–Basedow syndrome. After genetic diagnosis, two neurofibromas were found in the patient’s foot. In a 39-year-old female with heart palpitations as the principal symptom of a PCC, we identified the novel c.7330_7331insA (p.Thr2444AsnfsTer4) variant in heterozygosis. Another novel variant was noted in patient P23 with PCC and familiar NF. The variant consists of the insertion of TG in position 555 (c.555_556insTG; p.Asp186TrpfsTer6), resulting in a frameshift. We identified the same variant in the daughter of the patient who presented the same pathology. The last variant in the *NF1* gene was the novel c.5423C>T; p.Thr1808Met, mentioned before in patient P59.

#### 2.2.3. Genetic Variants Detected in RET

Three patients had variants in the *RET* gene (3/61, 4.9%), and all of them were missense and were identified in cases with PCC. The frequent variant c.2410G>A (p.Val804Met) was noted in a 70-year-old female with an isolated PCC (P39), while the c.2671T>G (p.Ser891Ala) variant was detected in a 43-year-old male (P40). The brother and daughter of this last patient were also carriers of the variant but are healthy at age 40 and 7, respectively. Both *RET* variants had been previously reported in medullary thyroid carcinoma (MTC) and/or MEN2 [39,40,47,48] but only the c.2410G>A (p.Val804Met) variant has been described in patients with isolated PCC [38]. We identified the VUS c.3149G>A; p.Arg1050Gln variant in exon 19 of the *RET* gene in patient P42. This variant has been detected in breast cancer [41]. This female was referred to a clinician due to headache and HTA during pregnancy at age 38 and a unilateral PCC.

#### 2.2.4. VHL Variants

Two missense variants, c.500G>A and c.599G>C, were found in the *VHL* gene (2/61, 3.3%). Patient P27, with a unilateral pheochromocytoma diagnosed at age 21 and a family history of PCC, carried the heterozygous c.500G>A (p.Arg167Gln) variant. Although this variant was first found in five unrelated individuals with Von Hippel–Lindau (VHL) disease [49], it was lately reported in isolated PCC [37]. The same heterozygous variant was also found in the father and brother of the index case, both with a diagnosis of PCC. The novel likely pathogenic variant c.599G>C (p.Arg200Pro) was noted in a patient (P32) diagnosed with familiar bilateral PCC. Moreover, the brother of the patient, also diagnosed with bilateral PCC, carries the same genetic change.

#### 2.2.5. Variants in MDH2

A missense VUS variant in *MDH2* (Malate Dehydrogenase 2) was detected in P2, a patient with a PCC diagnosed at age 58. Although this c.196G>A; p.(Ala66Thr) variant has been associated with severe encephalopathy [34], several other variants in *MDH2* have been reported in PPGLs that point to its pathogenicity [35,50,51].

#### 2.2.6. Molecular Diagnosis of Two Patients with PCC and Gonadal Dysgenesis

A 17-year-old female was referred to a clinician due to primary amenorrhea and pubertal delay. She had primary hypogonadism and was diagnosed with 46,XX gonadal dysgenesis. Fifteen years later, a left PCC was diagnosed, and she underwent surgery. Four years later, a right PCC was found, and she had surgery again. Her younger sister was diagnosed at age 7 with HBP and at age 14, she was diagnosed with 46,XY gonadal dysgenesis. When she was 18 years old, a left PCC was detected, and she underwent surgery. Both siblings presented the *CYP17A1* c.1246C>T; p.(Arg416Cys) variant in homozygosis, which has been previously associated with 17-alpha-hydroxylase deficiency [36] and explains the phenotype. Familiar testing showed that the mother, sister, and one niece were healthy carriers of the variant.

## 3. Discussion

Significant advances in the understanding of the genetics of PPGLs have occurred in the last decade and, currently, nearly 80% of all patients with PPGLs can be explained by genetic variants [8]. Almost 40% of the patients carry germline variants in one of the twenty-five known genes, while somatic changes in these same genes or others explain the additional 30 to 40% of the cases [8]. The frequency of the overall genetic changes and the specific germline and/or somatic level for each of the susceptible genes differs [9]. The development of novel sequencing techniques has decreased the cost and time consumption of the genetic analysis, mostly TGP, which is widely used because of its cost effectiveness and easy management. In this study, we have designed a TGP, including 16 susceptible genes, to test the presence of germline variants in a cohort of 63 PPGL patients. We found gene variants associated with the pathology in 16 patients (25.4%), a slightly higher diagnostic yield compared to previously performed studies (6–24%) [34,37,52,53,54].

In our cohort, variants in *SDHB* are the most frequent gene changes found (6.6%), as previously described in PPGLs [8,9]. We describe for the first time the novel c.595_604delinsGG; p.(Tyr199GlyfsTer20) variant in a patient presenting with a PGL of the organ of Zuckerkandl and a tumour in the carotid body (P58). Seventy percent of the patients with PGLs in the Zuckerkandl organ have a disease-causing variant in the *SDHB* or *SDHD* genes [55]. The risk of metastasis is higher in patients with SDHB gene variants [5]; interestingly, the only patient with metastasis and a positive molecular diagnosis carried an *SDHB* variant (P1). A single pathogenic variant in *SDHD* was identified in our cohort, in contrast with a higher frequency described in other studies (2–9%) [56].

Germline *NF1* gene variants were also found in our cohort, and two frameshift and one missense variants were identified for the first time. PPGLs related to NF1 appear in the fourth decade of life, usually, and are normally unilateral adrenal tumours. The risk of metastasis is up to 10% in these cases [12]. In our study, only two out of four patients positive for *NF1* variants had unilateral PCC and neurofibromatosis, and none developed metastatic disease. The pathology was diagnosed close to the age of 40 years in P7 and the daughter of P23, but patient P23 was diagnosed when he was 70 years old. On the other side, the other two patients, P11 and P59 with *NF1* variants, were suspected to have sporadic PPGLs. Similarly, other studies have described the presence of NF1 germline variants in PPGL patients without neurofibromatosis [35]. Some studies had already reported PPGLs carrying variants in *NF1* and another driver gene [35], which could explain the variable clinical phenotype observed. NF1-associated PPGLs are rarely extra-adrenal, as observed in our patient P59 with mediastinum PGL and a second variant in *SDHB*.

Germline variants in some driver genes, such as *VHL* and *RET*, are known as highly penetrant [35]. Regarding the *VHL* gene, variants result in the loss of regulation of the hypoxia-inducible factor, leading to tumour development and metastasis [27]. Variant frequency is estimated to be 4–7% in PPGLs [57]. In our cohort, we found two missense alterations in this gene (3.3%). The novel c.599G>C variant was recorded in P32 presenting with bilateral PCC at age 30. A variant in the same codon had been associated with Von Hippel–Lindau syndrome without PCC [58]. Furthermore, the patient presented elevated noradrenaline levels, which is a classical biochemical phenotype of *VHL* variant carriers [12]. Patient P27 with a PCC was noted to have in heterozygosis the c.500G>A; p. Arg167Gln variant, already described in nonsyndromic PCCs [43]. We cannot discard the development of the syndrome in both patients since PCCs are the first symptoms in up to 50% of individuals with VHL syndrome, and diagnosis is made during early adulthood (median age 29 years). Similarly, three patients with variants in *RET* present only with PCCs and no other clinical features of MEN2. Only 15% of the patients with MEN2 present with PCC as the first manifestation, and the mean age at diagnosis is 35 years old [12]. The patients with *RET* variants in this study were older at disease onset.

Up to now, only nine PPGL patients with *MDH2* variants have been reported (HGMD, by July 2024) [35,50,51,59,60]. In our cohort, we identified a single VUS *MDH2* variant in a patient with PCC diagnosed at age 58. Previous reports showed that metastatic PPGLs accounted for 33% (3/9 cases) in patients with *MDH2* variants; however, no evidence of metastasis has been reported until now in our patient. Despite the low prevalence and incomplete penetrance observed for *MDH2* variants [59], we believe that follow up should be considered until the identification of additional gene changes provides more information about its clinical importance.

Our results revealed that patients with PPGLs and a positive germline variant are younger at diagnosis, suggesting that younger patients should undergo genetic screening, especially PGLs. This idea is in line with previous studies in which paediatric patients or those under 30 years with PPGLs showed a higher molecular diagnosis rate [8].

In this study, we did not reach a specific genetic diagnosis in 47 patients (74.6%). We found no variants in some of the genes considered to be prevalent in PPGLs, such as *SDHA* (succinate dehydrogenase complex subunit A), *SDHC* (succinate dehydrogenase complex subunit C), and *MAX*, but neither is less frequently mutated, like *SDHAF2* (succinate dehydrogenase complex assembly factor 2), *EGLN1*, or *FH* (Fumarate Hydratase) [8]. The number of genetic cases without genetic diagnosis is high, presenting a challenge for research in molecular genetics. TGP sequencing is limited to known genes and exons, and due to the number and speed with which novel genes are identified, implementation of the panel is required.

In conclusion, the clinical and molecular characterization of a cohort of patients with sporadic PPGLs has led to the identification of germline variants in a susceptibility gene in almost 26% of the patients. We have identified novel variants in known genes, such as *NF1* and *SDHD*. This highlights the importance of genetic screening to every patient diagnosed with PPGL, with a special focus on the young. A longer follow up of patients with variants in genes associated with syndromic forms is recommended, as well as in positive relatives. Most of the patients remain without a molecular diagnosis. For those unexplained cases, extended TGP or whole exome/genome sequencing should be considered. The recognition of the aetiology allows the patient to have an adjusted follow-up as well as an effect on the genetic advice given to the families.

## 4. Materials and Methods

### 4.1. Study Design and Patients

In this study, we have included 63 patients with PPGLs from several Spanish hospitals. Clinicians provided data including tumour type, age at diagnosis, biochemical characterization, family history of PPGL or related tumours, and other relevant data. The local ethical committee approved this study (Cruces University Hospital, Spain, CEIC E20/08), and written informed consent was obtained from all participants and their family members.

### 4.2. Genetic Screening and In Silico Analysis

Genomic DNA was isolated from peripheral blood leukocytes using the MagPurix 12S system (Zinexts Life Science Corp., New Taipei City, Taiwan), and DNA purity and concentration were determined using a Qubit 2.0 fluorometer (Thermo Fisher Scientific, Waltham, MA, USA).

The TGP was designed using the Ion AmpliSeq™ Designer (Thermo Fisher Scientific) tool and contained 16 frequent genes associated with PPGLs (*RET*, *VHL*, *NF1*, *SDHA*, *SDHB*, *SDHC*, *SDHD*, *SDHAF2*, *TMEM127*, *MAX*, *KIF1B*, *EGLN1*, *MDH2*, *FH*, *IDH1*, and *IDH2*). Libraries were prepared according to the manufacturer’s instructions, and samples were sequenced using the Ion PGM platform (Thermo Fisher Scientific). Variants were filtered to include only those with a Phred-like score ≥30 and, therefore, were associated with a *p*-value < 0.001 and a Minor Allele Frequency (MAF) < 1% in a 1000 genome browser (https://www.internationalgenome.org/1000-genomes-browsers/index.html, accessed on 1 October 2024), and Exome Aggregation Consortium (ExAC) (https://gnomad.broadinstitute.org/, accessed on 1 October 2024). 

The molecular diagnosis of patients P24 and P25 was performed using a TGP containing genes related to anomalies of sex differentiation and development, as described elsewhere [61].

We predicted the possible effect of novel nonsynonymous variants on the structure and function of the protein using CADD (Combined Annotation Dependent Depletion), Polyphen-2 (Polymorphism Phenotyping v2), SNPs and Go, Panther (Protein ANalysis THrough Evolutionary Relationships), and the calibrated scores given by VarSome [62] for Revel (Rare Exome Variant Ensemble Learner), SIFT (scale-invariant feature transform), Provean (Protein Variation Effect Analyzer), mutation taster, and M-CAP (Mendelian Clinically Applicable Pathogenicity) (see Table S1). We classified genetic variants according to the recommendations of the ACMG [63] using VarSome [62]. We searched for previously reported clinical associations in the ClinVar and HGMD databases and the literature (e.g., PubMed). We verified the genetic variants identified with the TGP by PCR and sequencing using the BigDye Terminator v3.1 Sequencing Kit on the ABI 3130xl DNA sequencer system (Applied Biosystems, Waltham, MA, USA). When possible, patients’ first-degree relatives were tested likewise.

### 4.3. Statistical Analysis

Qualitative variables are expressed as frequencies and percentages, while non-parametric quantitative variables are presented as the median and interquartile range (IQR). A comparison between genetically positive and negative patients was performed using Student’s *t*-test or chi-square, as appropriate (IBM SPSS Statistics, version 29.0.0.0). The results were considered statistically significant when *p* ≤ 0.05.

## Figures and Tables

**Table 1:** Clinical characteristics and comparison between genetically positive and negative patients with pheochromocytoma and paraganglioma.

	Pheochromocytomas				Paragangliomas			
	All	Positive	Negative	*p*-Value	All	Positive	Negative	*p*-Value
**Number of samples**	49	12	37		14	4	10	
**Age at diagnosis (y)**	54.0 (39.0–63.0)	39.0 (30.0–50.5)	56.0 (48.2–64.5)	**0.01**	55.0 (47.2–65.7)	26.5 (24.0–33.2)	64.0 (60.5–69.0)	**<0.01**
**Tumour size (cm)**	4.6 (3.4–5.8)	6.2 (4.0–7.0)	4.3 (3.3–5.3)	NS	4.5 (2.3–5.7)	5.4 (4.7–6.6)	3.2 (1.4–4.7)	NS
**Gender (F/M, %)**	27/49, 55.1	7/12, 58.3	20/37, 54.0	NS	4/14, 28.0	2/4, 50.0	2/10, 20.0	NS

A *p*-value ≤ 0.05 was considered statistically significant and is presented in boldface. F, female; M, male; NS, non-significant; y, years.

**Table 2:** Clinical characteristics and genetic findings in patients with pheochromocytoma and paraganglioma. The genetic variants described for the first time associated with PPGLs are highlighted in boldface.

Patient	Gender	Age at Diagnosis (y)	Symptoms at Presentation	Location	Metanephrine (µg/day)	Normetanephrine (µg/day)	Tumour Size (cm)	Metastasis	Gene Variant
P1	Female	23	Paroxysmal HBP	Adrenal	Normal	2550	10.0	Yes	*SDHB*, c.286+1G>A [33]
P2	Male	58	Asymptomatic	Adrenal	6497	13,919	ND	No	***MDH2*, c.196G>A; p.(Ala66Thr) [34]**
P3	Male	73	HBP	Adrenal	Normal	499	7.5	No	
P4	Female	53	HBP	Adrenal	381	495	3.2	No	
P5	Male	62	Asymptomatic	Adrenal	Normal	563	4.0	No	
P6	Female	48	Asymptomatic	Adrenal	609	1140	2.5	No	
P7	Female	36	Asymptomatic	Adrenal	14,532	1974	7.0	No	*NF1*, c.586+1G>A [35]
P8	Male	45	Asymptomatic	Adrenal	Normal	732	3.6	No	
P9	Female	49	Palpitation	Adrenal	1554	2734	4.0	No	
P10	Female	73	HBP	Adrenal	13,495	3390	10.0	No	
P11	Female	39	Palpitation	Adrenal	399	1132	2.7	No	*NF1*, c.7330_7331insA; p.(Thr2444Asnfs*4)
P12	Male	49	Headache	Adrenal	1788	3150	5.0	No	
P13	Male	63	HBP	Adrenal	Normal	1710	4.5	No	
P14	Male	29	HBP	Adrenal	Normal	14,557	4.7	No	
P15	Male	55	Asymptomatic	Adrenal	980	Normal	3.1	No	
P16	Male	63	HBP	Adrenal	8460	2604	5.0	No	
P17	Female	39	HBP	Adrenal	Normal	4947	5.5	No	
P18	Female	41	HBP	Adrenal	Normal	3000	4.0	No	
P19	Female	45	Asymptomatic	Adrenal	2640	1417	4.7	No	
P20	Female	62	Asymptomatic	Adrenal	ND	ND	ND	No	
P21	Female	50	Paroxysmal HBP	Adrenal	1952	2302	4.7	Yes	
P22	Male	11	Paroxysmal HBP	Adrenal	ND	ND	ND	No	
P23	Male	70	ND	Adrenal	ND	ND	ND	No	*NF1*, c.555_556insTG; p.(Asp186Trpfs*6)
P24	Female	30	HBP	Adrenal	ND	ND	ND	No	*CYP17A1*, c.1246C>T; p.(Arg416Cys) [36]
P25	Female	17	HBP	Adrenal	ND	ND	5.5	No	*CYP17A1*, c.1246C>T; p.(Arg416Cys) [36]
P26	Female	ND	ND	Adrenal	ND	ND	ND	ND	
P27	Male	ND	ND	Adrenal	ND	ND	ND	ND	*VHL*, c.500G>A; p.(Arg167Gln) [37]
P28	Female	60	Mass effect	Adrenal	ND	ND	10.5	Yes	
P29	Female	54	Paroxysmal HBP	Adrenal	Normal	1670	ND	No	
P30	Female	ND	HBP	Adrenal	ND	ND	ND	No	
P31	Female	50	Asymptomatic	Adrenal	ND	ND	2.1	No	
P32	Female	30	NA	Adrenal	ND	ND	ND	No	***VHL*, c.599G>C; p.(Arg200Pro)**
P33	Male	66	Asymptomatic	Adrenal	445	1102	ND	No	
P34	Male	29	Paroxysmal HBP	Adrenal	7003	8442	4.9	No	
P35	Female	58	HBP	Adrenal	1026	Normal	6.7	No	
P36	Female	ND	Sweating	Adrenal	Normal	2369	5.5	No	
P37	Male	68	Asymptomatic	Adrenal	459	580	2.2	No	
P38	Female	57	HBP	Adrenal	1299	734	3.6	No	
P39	Male	68	Asymptomatic	Adrenal	Normal	1343	ND	No	*RET*, c.2410G>A; p.(Val804Met) [38]
P40	Male	43	Headache	Adrenal	60,129	24,023	7.0	No	*RET*, c.2671T>G; p.(Ser891Ala) [39,40]
P41	Male	76	HBP	Adrenal	668	Normal	2.5	No	
P42	Female	39	Paroxysmal HBP	Adrenal	5175	1368	3.5	No	***RET*, c.3149G>A; p.(Arg1050Gln) [41]**
P43	Female	55	Asymptomatic	Adrenal	3549	1321	ND	No	
P44	Male	61	Asymptomatic	Adrenal	16,603	3656	7.0	No	
P45	Male	77	Asymptomatic	Adrenal	1803	1213	4.2	No	
P46	Male	65	HBP	Adrenal	1383	Normal	2.4	No	
P47	Female	37	Palpitations	Adrenal	8430	13,755	7.6	No	
P48	Female	75	Hematuria	Adrenal	ND	ND	1.7	No	
P49	Male	74	Asymptomatic	Adrenal	863	599	4.2	No	
P50	Female	46	Mass effect	Abdominal	Normal	Normal	4.5	No	***SDHD*, c.52+1G>A**
P51	Male	24	Heart failure	Abdominal	819	Normal	4.8	No	*SDHB*, c.725G>A; p.(Arg242His) [37]
P52	Male	51	Mass effect	Abdominal	ND	ND	1.4	No	
P53	Male	56	Mass effect	Lumbar	ND	ND	ND	No	
P54	Male	66	Mass effect	Lumbar	ND	Normal	ND	No	
P55	Male	65	Mass effect	Abdominal	2269	668	ND	No	
P56	Female	70	Mass effect	Cervical	Normal	Normal	5.5	No	
P57	Female	76	Tinnitus	Cervical	ND	ND	0.5	No	
P58	Female	29	Palpitations	Abdominal	ND	ND	6.0	No	*SDHB*, c.595_604delinsGG; p.(Tyr199Glyfs*20)
P59	Male	24	Sweating	Mediastinum	2145	Normal	8.5	No	*SDHB*, c.72+1G>A [42]
									***NF1*, c.5423C>T; p.(Thr1808Met)**
P60	Male	60	Asymptomatic	Cervical	Normal	Normal	1.5	No	
P61	Male	78	Asymptomatic	Abdominal	620	Normal	7.0	No	
P62	Male	62	Asymptomatic	Abdominal	1092	Normal	4.0	No	
P63	Male	63	Mass effect	Cervical	Normal	Normal	3.2	No	

HBP, high blood pressure; ND, not determined; y, years. Sequence information is based on the following reference sequences: *CYP17A1*, NM_000102.4; *KIF1B*, NM_015074.3; *MDH2*, NM_005918.2; *NF1*, NM_001042492.3; *RET*, NM_020975.6; *SDHB*, NM_003000.3; *SDHD*, NM_003002.4; *VHL*, NM_000551.3.

**Table 3:** **Gene variants identified in the analysed patients with PCC or PGL.** The genetic variants described for the first time associated with PPGL are highlighted in boldface.

Patient	Chromosome Position	Gene Variant ^a^	dbSNP	ACMG Classification	Zygosity	Familiar Testing
P1	1:17359554	*SDHB*, c.286+1G>A [33]	rs786201063	P	Het	No
P2	7:75684277	***MDH2*, c.196G>A; p.(Ala66Thr)** [34]	rs141539461	VUS	Het	No
P7	17:29497016	*NF1*, c.586+1G>A [35]	rs1555607126	P	Het	Brother (wt)
P11	17:29677208	*NF1*, c.7330_7331insA; p.(Thr2444Asnfs*4)	rs1064794278	P	Het	No
P23	17:29496980	*NF1*, c.555_556insTG; p.(Asp186Trpfs*6)	ND	LP	Het	Daughter (het)
P24	10:104590740	*CYP17A1*, c.1246C>T; p.(Arg416Cys) [36]	rs1178684770	LP	Hom	Mother, sister, niece (het); niece (wt)
P25	10:104590740	*CYP17A1*, c.1246C>T; p.(Arg416Cys) [36]	rs1178684770	LP	Hom	Mother, sister, niece (het); niece (wt)
P27	3:10191507	*VHL*, c.500G>A; p.(Arg167Gln) [37]	rs5030821	P	Het	Father, brother (het)
P32	3:10191606	***VHL*, c.599G>C; p.(Arg200Pro)**	rs754016774	P	Het	Brother (het)
P39	10:43614996	*RET*, c.2410G>A; p.(Val804Met) [38]	rs79658334	P	Het	Son, sister (wt)
P40	10:43615592	*RET*, c.2671T>G; p.(Ser891Ala) [39,40]	rs75234356	P	Het	Mother (wt); brother, daughter (het)
P42	10:43622132	***RET*, c.3149G>A; p.(Arg1050Gln)** [41]	rs200956659	VUS	Het	No
P50	11:111957684	***SDHD*, c.52+1G>A**	rs1592777386	P	Het	Mother, sister, son, daughter (wt)
P51	1:17349143	*SDHB*, c.725G>A; p.(Arg242His) [37]	rs74315368	P	Het	No
P58	1:17350506	*SDHB*, c.595_604delinsGG; p.(Tyr199Glyfs*20)	rs1131691059	P	Het	Mother, aunt (het)
P59	1:17380442	*SDHB*, c.72+1G>A [42]	rs587782703	P	Het	No
	17:29654671	***NF1*, c.5423C>T; p.(Thr1808Met)**	rs760649828	VUS	Het	

Het, heterozygous; Hom, homozygous; LP, likely pathogenic; ND, not determined; P, pathogenic; VUS, variant of unknown significance; Wt, wild type. ^a^ Reference is indicated if a gene variant has been previously associated with a disease.

## Data Availability

The genetic data are stored on the servers of the Biobizkaia Health Research Institute. These data can also be accessed upon reasonable request according to ethical considerations and informed consent.

## Supplementary Materials

The supporting information can be downloaded at https://www.mdpi.com/article/10.3390/ijms252212056/s1.
