# Supplementary material for: Novel Gene Variants in a Nationwide Cohort of Patients with Pheochromocytoma and Paraganglioma
Source: Int J Mol Sci. 2024 Nov 9;25(22):12056. doi: 10.3390/ijms252212056 (PMC11593415; doi:10.3390/ijms252212056)
Supplement: Supplementary file 1 [file ijms-25-12056-s001.zip › ijms-3268863-supplementary.pdf]

Table S1. Gene variant characterization: Allele frequency and disease prediction by ACMG classification and by different in silico programs.

Patient	Gene variant	GnomAD (Overall/Specific[a])	ACMG classification	ClinVar	SIFT	Provean	Polyphen	Mutation Taster	Panther	SNPs and Go	M-CAP	CADD PHRED	REVEL	MobiDetails
P1	*SDHB*, c.286+1G>A [29]	ND/ND	P	P	ND	ND	ND	ND	ND	ND	ND	ND	ND	Alteration of the consensus splice site (98.41%)
P2	*MDH2*, c.196G>A; p.(Ala66Thr) [30]	0.0001274/0.0002358	VUS	VUS	VUS	VUS	B	VUS	Pdam	Dis	Bsupp	LB	Bmod	ND
P7	*NF1*, c.586+1G>A [31]	ND/ND	P	P	ND	ND	ND	ND	ND	ND	ND	ND	ND	ND
P11	*NF1*, c.7330_7331insA; p.(Thr2444Asnfs*4)	ND/ND	P	P	ND	ND	ND	ND	ND	Dis	ND	ND	ND	ND
P23	*NF1*, c.555_556insTG; p.(Asp186Trpfs*6)	ND/ND	LP	ND	ND	ND	ND	ND	ND	ND	ND	ND	ND	ND
P24	*CYP17A1*, c.1246C>T; p.(Arg416Cys) [32]	0.000005364/0.0	LP	P	Psupp	Pmod	Pdam	VUS	Pdam	Dis	Pmod	LB	Pmod	ND
P25	*CYP17A1*, c.1246C>T; p.(Arg416Cys) [32]	0.000005364/0.0	LP	P	Psupp	Pmod	Pdam	VUS	Pdam	Dis	Pmod	LB	Pmod	ND
P27	*VHL*, c.500G>A; p.(Arg167Gln) [33]	0.000003977/0.0	P	VUS	Psupp	VUS	Pdam	Bsupp	Pdam	Dis	Pmod	LB	Pmod	ND
P32	*VHL*, c.599G>C; p.(Arg200Pro)	ND/ND	P	VUS	Psupp	VUS	Pdam	Bsupp	Pdam	Dis	Pmod	LB	Pmod	ND
P39	*RET*, c.2410G>A; p.(Val804Met) [34]	0.0001364/0.0001373	P	P/LP	VUS	Bsupp	Pdam	VUS	Pdam	N	Psupp	LB	Psupp	ND
P40	*RET*, c.2671T>G; p.(Ser891Ala) [35,36]	0.00001197/0.00001575	P	P	Psupp	VUS	Pdam	VUS	Pdam	N	VUS	LB	Psupp	ND
P42	*RET*, c.3149G>A; p.(Arg1050Gln) [37]	0.00003183/0.00001756	VUS	VUS	VUS	Bmod	Pdam	VUS	Pdam	Dis	VUS	Ldel	Bsupp	ND
P50	*SDHD*, c.52+1G>A	ND/ND	P	P	ND	ND	ND	ND	ND	ND	ND	ND	ND	Alteration of the consensus splice site (98.41%)
P51	*SDHB*, c.725G>A; p.(Arg242His) [33]	0.00001193/0.0	P	P/LP	Psupp	VUS	Pdam	VUS	Pdam	Dis	Pmod	Ldel	Pmod	ND
P58	*SDHB*, c.595_604delinsGG; p.(Tyr199Glyfs*20)	ND/ND	P	P	ND	ND	Pdam	ND	Pdam	Dis	ND	ND	ND	ND
P59	*SDHB*, c.72+1G>A [38]	0.000004118/0.00001631	P	P	ND	ND	ND	ND	ND	ND	ND	ND	ND	ND
	NF1, c.5423C>T; p.(Thr1808Met)	0.00001193/0.00001574	VUS	VUS	Bsupp	VUS	B	B	ND	Dis	Pmod	LB	VUS	ND

B, benign; Bmod, benign moderate; Bsupp, benign supporting; Dis, disease; LB, likely benign; Ldel, likely deleterious; LP, likely pathogenic; N, neutral; ND, Not determined; P, pathogenic; Pdam, probably damaging; Pmod, pathogenic moderate; Possdam, possibly damaging; Psupp, pathogenic supporting; VUS, variant of unknown significance. [a]Specific allele frequency for the karyotype of the patient. Sequence information is based on the following reference sequences: *CYP17A1*, NM_000102.4; *MDH2*, NM_005918.2; *NF1*, NM_001042492.3; *RET*, NM_020975.6; *SDHB*, NM_003000.3; *SDHD*, NM_003002.4; *VHL*, NM_000551.3.
